# Fermented chrysanthemum stem as a source of natural phenolic compounds to alleviate tomato bacterial wilt disease

**DOI:** 10.1093/hr/uhaf027

**Published:** 2025-01-27

**Authors:** Peng Ren, Peijie Chen, Saisai Guo, Xinlan Mei, Gaofei Jiang, Tianjie Yang, Xiaofang Wang, Yangchun Xu, Qirong Shen, Zhong Wei

**Affiliations:** Key Lab of Organic-Based Fertilizers of China, Jiangsu Provincial Key Lab for Solid Organic Waste Utilization, National Engineering Research Center for Organic-Based Fertilizers, Jiangsu Collaborative Innovation Center for Solid Organic Waste Resource Utilization, Nanjing Agricultural University, Weigang 1, Nanjing 210095, China; Key Lab of Organic-Based Fertilizers of China, Jiangsu Provincial Key Lab for Solid Organic Waste Utilization, National Engineering Research Center for Organic-Based Fertilizers, Jiangsu Collaborative Innovation Center for Solid Organic Waste Resource Utilization, Nanjing Agricultural University, Weigang 1, Nanjing 210095, China; Key Lab of Organic-Based Fertilizers of China, Jiangsu Provincial Key Lab for Solid Organic Waste Utilization, National Engineering Research Center for Organic-Based Fertilizers, Jiangsu Collaborative Innovation Center for Solid Organic Waste Resource Utilization, Nanjing Agricultural University, Weigang 1, Nanjing 210095, China; Key Lab of Organic-Based Fertilizers of China, Jiangsu Provincial Key Lab for Solid Organic Waste Utilization, National Engineering Research Center for Organic-Based Fertilizers, Jiangsu Collaborative Innovation Center for Solid Organic Waste Resource Utilization, Nanjing Agricultural University, Weigang 1, Nanjing 210095, China; Key Lab of Organic-Based Fertilizers of China, Jiangsu Provincial Key Lab for Solid Organic Waste Utilization, National Engineering Research Center for Organic-Based Fertilizers, Jiangsu Collaborative Innovation Center for Solid Organic Waste Resource Utilization, Nanjing Agricultural University, Weigang 1, Nanjing 210095, China; Key Lab of Organic-Based Fertilizers of China, Jiangsu Provincial Key Lab for Solid Organic Waste Utilization, National Engineering Research Center for Organic-Based Fertilizers, Jiangsu Collaborative Innovation Center for Solid Organic Waste Resource Utilization, Nanjing Agricultural University, Weigang 1, Nanjing 210095, China; Key Lab of Organic-Based Fertilizers of China, Jiangsu Provincial Key Lab for Solid Organic Waste Utilization, National Engineering Research Center for Organic-Based Fertilizers, Jiangsu Collaborative Innovation Center for Solid Organic Waste Resource Utilization, Nanjing Agricultural University, Weigang 1, Nanjing 210095, China; Key Lab of Organic-Based Fertilizers of China, Jiangsu Provincial Key Lab for Solid Organic Waste Utilization, National Engineering Research Center for Organic-Based Fertilizers, Jiangsu Collaborative Innovation Center for Solid Organic Waste Resource Utilization, Nanjing Agricultural University, Weigang 1, Nanjing 210095, China; Key Lab of Organic-Based Fertilizers of China, Jiangsu Provincial Key Lab for Solid Organic Waste Utilization, National Engineering Research Center for Organic-Based Fertilizers, Jiangsu Collaborative Innovation Center for Solid Organic Waste Resource Utilization, Nanjing Agricultural University, Weigang 1, Nanjing 210095, China; Key Lab of Organic-Based Fertilizers of China, Jiangsu Provincial Key Lab for Solid Organic Waste Utilization, National Engineering Research Center for Organic-Based Fertilizers, Jiangsu Collaborative Innovation Center for Solid Organic Waste Resource Utilization, Nanjing Agricultural University, Weigang 1, Nanjing 210095, China

## Abstract

Natural antimicrobial compounds (NACs) in the plant stem are crucial for replacing conventional synthetic pesticides in the control of soil-borne diseases, and microbial fermentation can enhance their concentration and bioactivity. In this study, the stems of 10 plant species were collected for fermentation by probiotic bacteria *Bacillus amyloliquefaciens* T-5 to identify the most effective plant resource for controlling tomato bacterial wilt disease and discover key NACs. Chrysanthemum stem was identified as an optimal fermentation substrate, as its water-soluble extracts (WSEs) significantly inhibited the growth of pathogenic *Ralstonia solanacearum* and effectively alleviated tomato wilt under greenhouse conditions. Key metabolites, primarily phenolic acids including 2-hydroxy-3-phenylpropanoic acid (PLA), 3-(4-hydroxyphenyl)-propionic acid (HPPA), and mandelic acid (MA), were determined by metabolomics, all of which significantly inhibited the growth of *R. solanacearum* at a concentration of 0.2 mM, with only HPPA effectively controlling tomato wilt. Thus, fermented chrysanthemum stem contains NACs that are effective against bacterial wilt, providing a green option for controlling soil-borne diseases.

## Introduction

*Ralstonia solanacearum*, the causative agent of bacterial wilt disease, is a destructive pathogen with a wide host range, infecting >200 plant species, including many important horticultural crops such as tomato [1, 2]. According to the data from Our World in Data, China, India, Turkey, and the USA were the top four tomato-producing countries in 2022, with a production of 68.24, 20.69, 13.00, and 10.20 million tons, respectively (https://ourworldindata.org/grapher/tomato-production), highlighting the significant economic value of tomato. However, the outbreak of bacterial wilt disease results in yield losses ranging from 15% to 95%, which becomes a limiting factor for agricultural production and economic development [3]. While plant pathogens can be treated with pesticides, the intensive use of these chemicals has led to the emergence of resistant microbial pathogens causing serious problems for human health and environmental quality [4, 5].

Biodegradable NACs have recently reemerged as a promising alternative to conventional chemical pesticides. For example, plant NACs, including phenolic acids, flavonoids, saponins, and terpenes [6], more commonly known as plant secondary metabolites (SMs), have excellent pharmacological activities and play crucial roles in the control of soil-borne diseases [7]. Rongai et al. reported that pomegranate peel extract could reduce the density of the soil pathogen *Fusarium oxysporum*, with HPLC-DAD-ESI/MS analysis identifying the main antimicrobial components as punicalagin and ellagic acid [8]. Similarly, Ozkaya et al. also reported that garlic extract obtained by ethanol extraction significantly reduced diseases of tomato and rice [9]. Although these methods are effective in preventing and controlling plant diseases, solvent extraction, particularly organic solvents, tends to have the disadvantages of high consumption, solvent residues, and harmful effects on humans and the environment [10], which pose challenges for environmental sustainability and emphasize the need for greener methods to obtain NACs.

Microbial fermentation of plant tissues, such as stem, root, and leaf, offers a safer and more eco-friendly alternative, as NACs are released during lignocellulose degradation by hydrolytic enzymes secreted by microorganisms [11–13]. For example, the contents of quercetin and kaempferol in *Lespedeza cuneata* significantly increased by probiotic fermentation compared with nonfermentation [14]. A co-fermentation strategy involving *Monascus anka* and *Saccharomyces cerevisiae* enhanced the free flavonoid content in *Pericarpium Citri Reticulatae* by 48.12%, with various hydrolytic enzymes playing a crucial role in this process [15]. Moreover, microbial fermentation can enhance NACs solubility by biotransformation. As reported by Koirala, 7-O-phosphate, a derivative produced from naringenin catalyzed by *Bacillus amyloliquefaciens*, exhibited enhanced water solubility by ~45 times [16]. Microbial fermentation has become a popular approach for obtaining NACs [17]. However, studies have largely focused on the fermentation process, overlooking the effect of plant heterogeneity on NACs discovery.

NACs exploration is complex and challenging because of plant heterogeneity, which leads to considerable variations in NACs concentration and pharmacological activity [18]. For instance, chemical analyses by Đorđević et al. revealed that wheat stem contained higher levels of phenolic acids and flavonoids than corn stem, contributing to enhanced antioxidant activity [19]. Another study reported that essential oils from eucalyptus and rosemary exhibited distinct inhibitory effects on the growth of *Penicillium expansum*, a pathogenic fungus that infects apple and pear, thereby leading to reduced fruit yields [20]. In addition, different plants synthesize specific NACs, such as artemisinin in *Artemisia annua* L*.* and paclitaxel in *Taxus brevifolia* Nutt*.*, which play crucial roles in disease treatment because of their potent therapeutic effects [21]. These variations underscored the necessity to broaden our research scope to include a wide array of plant species, thereby enhancing the discovery of high-value NACs. Fortunately, horticultural management and agricultural production provide sufficient plant resources for NACs discovery.

**Figure 1:**
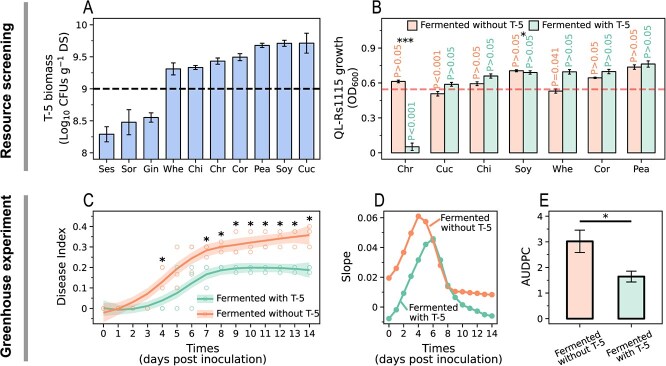
Results of resources screening and greenhouse experiments. (**A, B**) Biomass of strain T-5 (**A**) and the OD_600_ at 48 h of strain QL-Rs1115 (**B**) were examined to characterize the ability of strain T-5 to utilize stems and the suppression effect of WSEs of stems fermented with or without strain T-5 on pathogen *in vitro*. The dashed line in **A** represents the CFUs threshold below which fermentation combinations were excluded. The dashed line in **B** indicates the blank control, representing the mean OD_600_ value for strain QL-Rs1115 growth at 48 h under conditions where an equal volume of sterile water replaced WSEs. *P*-values on each bar indicate the significance level, showing whether the ‘Fermented with T-5’ or ‘Fermented without T-5’ treatment was significantly less than the blank control, and the asterisks indicate whether ‘Fermented with T-5’ was significantly less than ‘Fermented without T-5’. (**C**–**E**) Effects of WSE of fermented chrysanthemum stem on disease progress curve (**C**), slope of disease progress curve (**D**), and AUDPC (**E**) of tomato based on greenhouse experiments. In **C**, the curves represent the nonlinear fitting between times and disease indices based on the ‘loess’ method. In **D**, the gradient of each time point was taken as the slope. ‘^*^’ and ‘^***^’ denote significant differences at *P* < 0.05 and *P* < 0.001, respectively. All *P*-values were determined by one-tailed Mann–Whitney *U* tests.

Here, we selected 10 plant resources (wheat, corn, soybean, peanut, chili, chrysanthemum, cucumber, sorghum, ginger, and sesame) and used *B. amyloliquefaciens* T-5, a beneficial bacterium screened from the tomato rhizosphere, for fermentation of their stems. This study aimed to identify the optimal plant material whose fermentation products can protect tomato against *R. solanacearum* and to pinpoint the key NACs responsible for this effect. Initially, the inhibitory effects of water-soluble extracts (WSEs) of fermented plant stems on *R. solanacearum* were evaluated, while efficacy to control tomato wilt was investigated under greenhouse conditions based on the optimal plant material. Moreover, these NACs were identified by metabolomic analysis and validated for their efficacy under both laboratory and greenhouse settings, ensuring the practical application of the findings.

## Results

### Chrysanthemum stem fermented by *B. amyloliquefaciens* strain T-5 can suppress pathogen growth and alleviate tomato wilt disease

To ensure effective fermentation, the colony-forming units (CFUs) of strain T-5 were assessed at the end of the fermentation process first. The biomass of T-5 postfermentation consistently fell <10^9^ CFUs per gram of dry stem for sesame, ginger, and sorghum (Fig. 1A). Hence, the stems of chili, wheat, chrysanthemum, peanut, cucumber, corn, and soybean were retained for further analysis. Subsequently, we proceeded to identify the most effective resources from the stems of these seven plants. Compared with WSEs of stems fermented without strain T-5 and the blank control, only WSE of chrysanthemum stem fermented with strain T-5 exhibited the most significant inhibitory effect on the growth of strain QL-Rs1115 (Fig. 1B; *P* < 0.001, one-sided Mann–Whitney *U* test). In addition, no significant correlation was found between the CFUs of strain T-5 and the growth of pathogen QL-Rs1115, indicating that the observed inhibitory effects were not a result of T-5 biomass (Fig. S1; *P* > 0.05).

**Figure 2:**
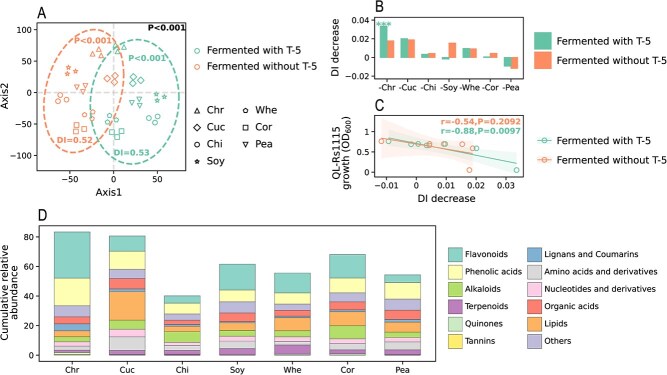
Analysis of plant stem metabolomic. (**A**) T-distributed stochastic neighbor-embedding analysis of metabolomics for different treatments. The `P' and `DI' inside the ellipse represent the significance and divergence index within the system, while the `P' outside the ellipse represents the significance between systems. All *P*-values were obtained by PERMANOVA (permutations = 999). (**B**) DI decrease after removing each stem from its system, and ‘^***^’ denotes significant difference at *P* < 0.001 based on the one-tailed Mann–Whitney *U* test. (**C**) Correlation analysis between DI decrease and strain QL-Rs1115 growth. ‘r’ and ‘*P*’ represent Pearson correlation coefficient and significance, respectively. (**D**) Cumulative relative abundance of metabolites in fermented stems of different plants. Before calculating the cumulative relative abundance, the relative abundance of each metabolite was standardized so that the sum of its relative abundance in all samples was 1.

We then explored the control effect of WSE of fermented chrysanthemum stem on tomato wilt [22]. The results of greenhouse experiments showed that the disease progress curve based on the disease index exhibited an overall S-shaped pattern, and adding WSE of fermented chrysanthemum stem significantly reduced the disease index on Day 4 and Days 7–15 (Fig. 1C; *P* < 0.05, one-sided Mann–Whitney *U* test). Rate and occurrence time of disease were also reduced and delayed (Fig. 1D). Furthermore, we calculated the area under the disease progress curve (AUDPC) for each treatment. AUDPC of tomato treated with WSE of chrysanthemum stem fermented with strain T-5 was significantly lower than that of tomato treated with WSE of chrysanthemum stem fermented without strain T-5 (Fig. 1E; *P* < 0.05, one-sided Mann–Whitney *U* test).

These findings collectively suggested that chrysanthemum stem was an optimal plant material, whose fermentation products inhibited *R. solanacearum* growth and significantly alleviated tomato wilt, and contained key NACs released after microbial fermentation. However, these specific antibacterial compounds still needed to be further explored based on metabolomics.

### Fermented chrysanthemum stem exhibits the highest chemical variation and antibacterial effect

The strong antibacterial effect of fermented chrysanthemum stem on *R. solanacearum* indicated there may be a high variation in its chemical composition. Thus, stems fermented with and without strain T-5 were considered as two independent metabolomics systems to calculate the divergence index (DI), a metric for estimating plant residue chemical [23]. A significant difference was found between the two systems, while an increase in DI was observed in the fermentation system (Fig. 2A; *P* < 0.001, permutational multivariate analysis of variance [PERMANOVA]). We then calculated the DI decrease by removing each stem from its metabolomics system. Removing the fermented chrysanthemum stem significantly decreased DI of the system (Fig. 2B; *P* < 0.001, one-sided Mann–Whitney *U* test), thereby stabilizing the system and highlighting its high chemical variation and differences. In addition, DI decrease in the fermentation system was significantly negatively correlated with strain QL-Rs1115 growth (Fig. 2C; *P* < 0.01). Subsequently, the categories and proportions of metabolites were analyzed, and the results showed that flavonoids (22.24%) and phenolic acids (16.83%) were the main metabolites (Fig. S2). Interestingly, the cumulative relative abundance of these compounds was higher in fermented chrysanthemum stem than in other stems (Fig. 2D), suggesting their role in affecting the chemical variation of chrysanthemum stem.

### A subset of phenolic acid-dominant differential metabolites influences the chemical variation and antibacterial effect of fermented chrysanthemum stem

**Figure 3:**
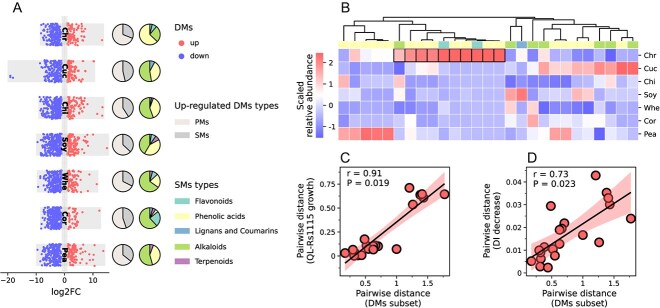
Analysis of DMs. (**A**) Volcano plot of DMs. ‘up’ and ‘down’ correspond to metabolites significantly upregulated and downregulated in stems fermented with strain T-5 compared with stems fermented without strain T-5. The two columns of pie charts on the right show the proportions of PMs and SMs, as well as SM types, in upregulated DMs. (**B**) Relative abundances of significantly upregulated SMs from fermented chrysanthemum stem and fermented stems of different plants. In **B**, the relative abundance of each metabolite in all fermented stems was scaled using the *z*-score method. Metabolites marked with black border lines were a DM subset with the highest relative abundance in fermented chrysanthemum stem. The color stripe between the clustering dendrogram and the heat map represents the classification of each metabolite. (**C**) Mantel correlation between the DM subset and DI decrease (permutations = 999). (**D**) Mantel correlation between the DM subset and QL-Rs1115 growth (permutations = 999). In **C** and **D**, Bray–Curtis distance was used to calculate the pairwise distance of the DM subset, while Euclidean distance was used to determine DI decrease and QL-Rs1115 growth.

We found that fermentation significantly altered the metabolomic of each stem (Fig. S3; *P* < 0.05, PERMANOVA). Differential metabolites (DMs), determined by variable importance in projection (VIP) extracted from the orthogonal partial least squares-discriminant analysis (OPLS-DA) model, *P* (corrected using the false discovery rate [FDR]), and fold changes (FC), were analyzed between each stem fermented with and without strain T-5, with red and blue points representing significantly upregulated (VIP > 1, *P* < 0.01, log_2_FC > 1) and downregulated (VIP > 1, *P* < 0.01, log_2_FC < 1) DMs after fermentation, respectively (Fig. 3A). Although the primary metabolites (PMs) were predominant among the upregulated DMs, SMs such as phenolic acids, flavonoids, alkaloids, and terpenoids are crucial for pharmacological activity [6]. Thus, we focused on the significantly upregulated SMs in fermented chrysanthemum stem and found that phenolic acids had the highest proportion (Fig. 3A, Table S2). Moreover, only two SMs were shared across all fermented stems, reflecting the inherent heterogeneity among plant species (Fig. S4). Ten SMs, predominantly phenolic acids, followed by flavonoids and alkaloids, exhibited the highest relative abundance in fermented chrysanthemum stem, showing distinct patterns compared with other fermented stems (Fig. 3B, Fig. S5), and they were thus considered a DM subset. Notably, the relative abundance of the DM subset significantly correlated with both DI decrease and pathogen growth (Fig. 3C and D), suggesting their substantial influence on the chemical variation and antibacterial effect of chrysanthemum stem fermented with strain T-5, as well as their crucial roles.

### Three phenolic acids are key metabolites in fermented chrysanthemum stem

**Figure 4:**
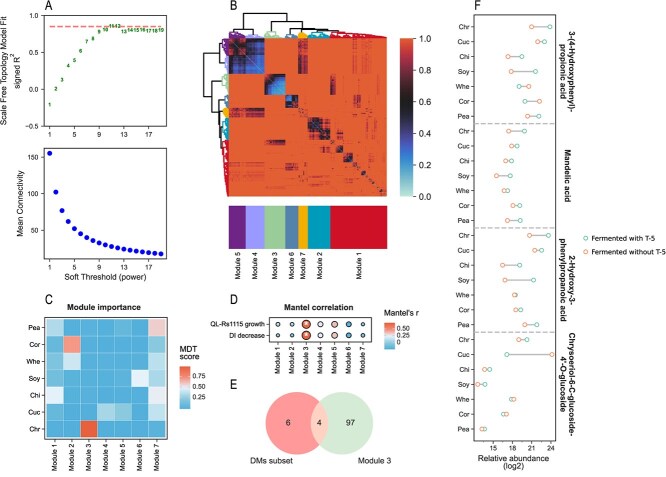
Metabolomic WGCNA. (**A**) Soft-threshold screening of WGCNA based on R^2^ of scale-free topology model fit and mean connectivity under different soft thresholds. The dashed line represents the threshold (0.85). (**B**) Dissimilarity topological overlap matrix plot and module division. (**C**) Evaluation of module importance based on MDT. (**D**) Mantel correlation between modules and traits. ‘^*^’ denotes significant difference at *P* < 0.05 (permutations = 999). (**E**) Intersection of the key Module 3 and significantly upregulated DM subset from fermented chrysanthemum stem. (**F**) Relative abundance of metabolites from the intersection of **E**.

Our analysis confirmed that the chemical variation of fermented chrysanthemum stem played a crucial role in its antibacterial effects, with a DM subset, primarily phenolic acids, influencing both chemical variation and antibacterial activity. However, the specific key compounds remained unclear. To address this, weighted gene coexpression network analysis (WGCNA) was used to divide metabolites into different modules and identify key metabolites. First, hierarchical clustering was performed on all fermented samples (*n* = 21) to identify and exclude any abnormal samples that might affect WGCNA [24], and the results showed that all samples met the analysis requirements (Fig. S6A). Next, metabolites with minimal variation across all samples were removed, and the remaining metabolites were used for further analysis (Fig. S6B; *P* > 0.01, Kruskal–Wallis test). The results revealed that 11 was the best soft threshold, with R^2^ of scale-free topology model fit >0.85 and a small mean connectivity (Fig. 4A; R^2^ = 0.8518, mean connectivity = 27.69). The metabolites were divided into seven modules (Fig. 4B), each containing different metabolites (Fig. S7). We then used a multivariate decision tree (MDT) to evaluate the importance of each module, revealing that Module 3 had the highest MDT score in fermented chrysanthemum stem (Fig. 4C, Table S3). In addition, Module 3 was significantly correlated with DI decrease and QL-Rs1115 growth, with Mantel correlation coefficients of 0.69 and 0.65, respectively (Fig. 4D; *P* < 0.05, permutations = 999), indicating its status as a key module for fermented chrysanthemum stem. Finally, by intersecting Module 3 and the significantly upregulated DM subset in fermented chrysanthemum stem, we identified four key metabolites (mainly phenolic acids), including 2-hydroxy-3-phenylpropanoic acid (PLA), mandelic acid (MA), 3-(4-hydroxyphenyl)-propionic acid (HPPA), and chrysoeriol-6-C-glucoside-4’-O-glucoside (Fig. 4E and F; Table S4). Interestingly, these metabolites exhibited high relative abundance in stems fermented without strain T-5, suggesting that they were plant NACs.

### HPPA can protect tomato against *R. solanacearum*

**Figure 5:**
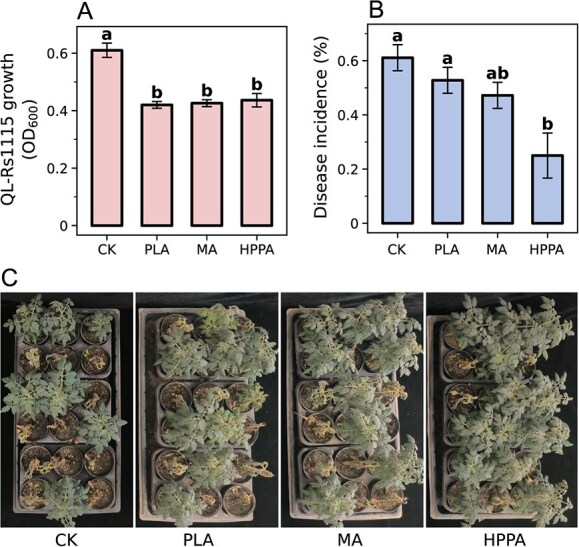
Effect of key metabolites on pathogen growth and bacterial wilt control. (**A**) Effect of metabolites at a concentration of 0.2 mM on the growth of strain QL-Rs1115. (**B**) Effect of metabolites at the concentration of 0.2 mM on the incidence of tomato wilt. (**C**) Greenhouse experiments. CK: blank control; PLA: 2-hydroxy-3-phenylpropanoic acid; HPPA: 3-(4-hydroxyphenyl)-propionic acid; MA: mandelic acid. In **A** and **B**, lowercase letters above the bars (mean + SD) denote statistically significant differences based on Dunn’s test (*P* < 0.05).

We highlighted the potential of SMs (mainly phenolic acids) from fermented chrysanthemum stem in inhibiting pathogenic *R. solanacearum* growth and reducing tomato wilt disease. To further verify the effects of these compounds, we evaluated their inhibitory capacity against strain QL-Rs1115 growth *in vitro*. The results revealed that 0.2 mM of PLA, MA, and HPPA significantly inhibited the growth of strain QL-Rs1115 with similar levels of antibacterial activity compared with the control, as indicated by OD_600_ values of 0.4225, 0.4262, and 0.4386, respectively, at 48 h (Fig. 5A; *P* < 0.05, Dunn’s test). However, because *in vitro* inhibition does not guarantee disease control efficacy, greenhouse experiments were performed. The results suggested a reduction in the incidence of tomato wilt from 61.11% in the control group to 52.78%, 47.22%, and 25.00% in the PLA, MA, and HPPA treatment groups, respectively. Among these, only HPPA was statistically significant, confirming its superior effectiveness in controlling bacterial wilt in tomato (Fig. 5B and C; Fig. S8; *P* < 0.05, Dunn’s test).

Overall, these findings support the potential of phenolic acids, particularly HPPA, as NACs for tomato disease control. The results of *in vitro* and greenhouse experiments underscored the value of HPPA in developing eco-friendly solutions for managing tomato bacterial wilt. Future research should focus on assessing the stability and efficacy of HPPA under field conditions and exploring its broader agricultural applications.

## Discussion

We investigated the potential of plant stems fermented by strain T-5 to produce NACs for protecting tomato against *R. solanacearum*, the causative agent of bacterial wilt. The results showed that fermented products from chrysanthemum stem significantly inhibited *R. solanacearum* growth and alleviated tomato bacterial wilt disease. HPPA, a natural phenolic compound, was determined to be a key metabolite based on metabolomic and *in vitro* validation experiments. Application of HPPA or WSE of fermented chrysanthemum stem can be an eco-friendly alternative to conventional chemical pesticides.

The rapid evolution of *R. solanacearum* and its widespread dissemination have made NACs discovery a persistent topic [3, 25]. Given the negative impact of pesticide residue on the ecosystem, scientists are increasingly optimistic about NACs, with their properties of biodegradability and bioactivity. Plant NACs such as lansiumamide B, flavonoids, and essential oils could effectively inhibit the growth of *R. solanacearum*, indicating their potential as alternatives to conventional chemical pesticides [26–28]. Plant residues, such as stems, generated from horticultural and agricultural production offer diverse biomass resources for the NACs discovery, which is a win–win strategy for the prevention and control of soil-borne bacterial wilt and the recycling of waste. Moreover, the remarkable ability of microbial fermentation to enhance both concentration and bioactivity of NACs has made it an efficacious and competitive technology [17].

The objective of the present study was to determine a plant material whose fermented products could inhibit *R. solanacearum* growth and control tomato wilt. From the stems of 10 plants, chrysanthemum stem emerged as the optimal plant material, as its WSE fermented by *B. amyloliquefaciens* T-5 significantly inhibited *R. solanacearum* growth, indicating that fermented chrysanthemum stem may contain specific or higher NACs concentrations. Moreover, the addition of WSE of fermented chrysanthemum stem significantly reduced the incidence of tomato bacterial wilt. However, attributing the reduction in bacterial wilt solely to the direct inhibition of pathogens by WSE may be inaccurate, since WSEs may also play a role in regulating the rhizosphere microecosystem, which is a natural defense against *R. solanacearum* invasion [29]. While the change in the microbiome of the rhizosphere could be explored in future research, our results were closely related to plant stem chemistry.

The study further analyzed the metabolomic changes in fermented stems of different plants. Fermented chrysanthemum stem exhibited the highest chemical variation (evaluated by DI decrease), which strongly correlated with the inhibition of *R. solanacearum* growth (Fig. 2B and C). This chemical variation may be associated with flavonoids and phenolic acids, with the highest relative abundance detected in fermented chrysanthemum stem (Fig. 2D). Previous reports have also pointed out that chrysanthemum contains abundant flavonoids and phenolic acids [30]. DM analysis revealed that a subset of DMs, primarily phenolic acids, had the highest relative abundance in fermented chrysanthemum stem and were significantly correlated with DI decease and *R. solanacearum* growth (Fig. 3C and D), indicating that they were the main factors responsible for the variation and contained key metabolites. To identify these key metabolites, WGCNA, a technique commonly used in transcriptome analysis and increasingly applied to metabolomics [31–33], was used to divide metabolites into different modules. Ultimately, four compounds obtained from the intersection of the key module and DM subset were retained as key metabolites; these compounds were PLA, MA, HPPA, and chrysoeriol-6-C-glucoside-4’-O-glucoside. Among them, chrysoeriol-6-C-glucoside-4’-O-glucoside, a glycosylated flavonoid derived from chrysoeriol with antagonistic effect on various Gram-negative and Gram-positive bacteria [34], may have antimicrobial activity because glycosylation can enhance solubility and biological activity [35, 36]. PLA and HPPA have been shown to exhibit inhibitory effects on the pathogens causing black spot rot in apple and anthracnose in red pepper, respectively [37, 38], while MA is known for its broad-spectrum antimicrobial activity [39, 40].

Here, we focused on three phenolic acids representing the major categories of the significantly upregulated DM subset in fermented chrysanthemum stem and with the highest relative abundance (Fig. 3B). Previous research has demonstrated the antibacterial properties of these phenolic acids, but their effects on inhibiting the growth of the pathogen *R. solanacearum* and controlling tomato wilt have been largely unexplored. In verification experiments, all three phenolic acids significantly inhibited the growth of *R. solanacearum*, but only HPPA effectively controlled tomato wilt. A reasonable explanation for this outcome is that PLA and MA are α-hydroxy acids, which can be more readily used as carbon sources by most environmental microorganisms, potentially diminishing their efficacy in disease control [41, 42]. Here, HPPA is recommended as a NAC for controlling tomato wilt, as it is more likely a plant-derived SM because of its high relative abundance in stems fermented without strain T-5 (Fig. 4F). The annotation results from antiSMASH [43] support this hypothesis, as HPPA was not present in the predicted SMs of strain T-5 (Table S5).

In summary, from the perspective of plant heterogeneity, our study demonstrated that chrysanthemum stem fermented by *B. amyloliquefaciens* T-5 significantly alleviated tomato wilt and HPPA was the key compound. It also established a more accurate and detailed relationship between the utilization of plant residues and disease control. Furthermore, by meta-analysis and machine learning, we can achieve precise matching of plant resources and the controlling of soil-borne diseases, eventually discovering more NACs for managing various plant diseases. We hope that the findings of this study will inspire further research on natural products and contribute to the advancement of green agriculture and sustainable development.

## Conclusion

We explored the potential of various plant stem resources and microbial fermentation to determine effective NACs for controlling soil-borne bacterial wilt in tomato caused by *R. solanacearum.* Our research demonstrated that chrysanthemum stem, fermented with *B. amyloliquefaciens* T-5, emerged as the optimal substrate whose WSE exhibited significant inhibition of *R. solanacearum* growth, as well as reduction in the incidence of tomato wilt. Using metabolomics, we demonstrated heterogeneity among plants and identified three phenolic acids as key metabolites. Among these phenolic acids, HPPA proved particularly effective in controlling tomato wilt. Our findings underlined the importance of considering plant heterogeneity and provided two viable alternatives for controlling tomato wilt: using WSE of fermented chrysanthemum stem and HPPA as a natural pesticide. Future research should focus on a comprehensive evaluation of these methods to further support and enhance sustainable agricultural practices.

## Materials and methods

### Plant stem collection and preparation

We collected 10 plants, including wheat (Whe), corn (Cor), soybean (Soy), peanut (Pea), chili (Chi), chrysanthemum (Chr), cucumber (Cuc), sorghum (Sor), ginger (Gin), and sesame (Ses). After removing the roots and leaves, the stems were washed with deionized water and air-dried. Subsequently, the stems were ground using a grinder and sieved through a 20-mesh sieve (0.85 mm pore size) for further use.

### Microorganisms and culture conditions

*Bacillus amyloliquefaciens* T-5 and *R. solanacearum* QL-Rs1115, previously isolated from the tomato rhizosphere, were used in this study [44, 45]. Nutrient broth (NB) medium (10.0 g of glucose, 5.0 g of tryptone, 0.5 g of yeast extract, and 3.0 g of beef extract in 1000 ml of water; pH 7.0) was used to culture strains T-5 and QL-Rs1115 at 30°C and 170 rpm for 24 h to obtain microbial seed solutions in a 150-ml shake flask. To remove the culture medium, 2 ml of microbial seed solution was transferred to sterile 2-ml centrifuge tubes, centrifuged at 6000 rpm for 4 min, the supernatant was discarded, and the seed solution was resuspended in 2 ml sterile water. This process was repeated three times, and the optical density of the seed solutions at 600 nm (OD_600_) was measured using a multifunctional microplate reader (Infinite E Plex, Tecan, Austria) and adjusted to 0.5 for inoculation.

### Fermentation of plant stems

We used a small-scale fermentation system, which allows for more precise control over the fermentation process and mitigates the risk of contamination. Two fermentation modes including ‘Fermented with T-5’ (inoculated with strain T-5 seed solution) and ‘Fermented without T-5’ (inoculated with equal volume of sterile water) were set up, and the procedures were as follows: 0.8 g of plant stem powder was weighed into a 50-ml Falcon tube sealed with a vented sealing film with a 0.22-μm filter, and all tubes were sterilized in an autoclave (115°C for 30 min) to avoid contamination [46]. Water content of each tube was adjusted to 70% using mineral media (3.0 g of KH_2_PO_4_, 3.0 g of NaNO_3_, 0.5 g of CaCl_2_, 0.5 g of MgSO_4_·7H_2_O, 7.5 mg of FeSO_4_·7H_2_O, 2.5 mg of MnSO_4_·H_2_O, 2.0 mg of ZnSO_4_, 3.0 mg of CoC1_2_, and 1000 ml of water) and strain T-5 seed solutions or equal volume of sterile water was added at 1% (v/w) for fermentation. All the 50-ml Falcon tubes (10 stems × 2 fermentation modes × 3 replicates) were incubated at 30°C for 7 days with 80% relative humidity. After fermentation, 2.0 g of stem powder was weighed and transferred to a new 50-ml Falcon tube, and 12 ml of sterile water was added (1:6, w/v). The mixture was shaken for 1 h at 170 rpm, followed by 30-min rest. The supernatant was then collected as WSE for use in subsequent experiments.

### Strain T-5 biomass determination and inhibitory effect of WSEs on pathogen QL-Rs1115

#### Strain T-5 biomass determination

The biomass of strain T-5 in WSEs was enumerated using the dilution plating method on V8 selective medium [47], with incubation at 37°C for 36 h. CFUs were used to quantify the biomass of strain T-5, which was then converted to CFUs of strain T-5 per gram of dry stem (CFUs·g^−1^ DS).

#### Inhibitory effect of WSEs on pathogen QL-Rs1115

Sterile WSEs of plant stems fermented with or without strain T-5 were obtained by filtration through a sterile 0.22-μm syringe filter (Sigma–Aldrich, St. Louis, MO, USA). We then added 20 μl of sterile WSE (equal volume of sterile water as a blank control), 2 μl of the seed solution of strain QL-Rs1115, and 178 μl of 10% NB medium to each well of a 96-well microplate (Costar® 96-well cell culture plate; Corning, Glendale, AZ, USA). The microplate was then incubated at 30°C with 170 rpm, and the OD_600_ was measured after 48 h to characterize the growth of strain QL-Rs1115.

### Greenhouse experiments

Tomato seeds of cultivar ‘Jiangsu’ were first immersed in 70% (v/v) ethanol for 3 min, then immersed in 5% (v/v) sodium hypochlorite for 5 min, and then rinsed with sterile distilled water for three times to disinfect the surface of tomato seeds. These surface-disinfected tomato seeds were germinated on a water agar plate for 3 days, and then seeded into a seedling plate containing seedling substrate (Jiangsu Xingnong Substrate Technology Co., Ltd., Zhenjiang, Jiangsu, China). At the three-leaf stage, the germinated tomato plants were transplanted into seedling containers containing 100 g growth substrate (Jiangsu Xingnong Substrate Technology Co., Ltd.).

One week after transplantation, tomato plants were inoculated with strain QL-Rs1115 at a final concentration of 10^6^ CFUs·g^−1^ growth substrate. WSE (filtered through a sterile 0.22-μm syringe filter) derived from the plant stem fermented with strain T-5 was diluted 10 times with sterile water, and 10 ml of this solution was added to tomato plants by root drenching 3 days later. An equal volume of WSE obtained from the plant stem fermented without strain T-5 was used as a control. Each treatment was replicated three times, with 12 tomato plants in each replicate, resulting in 36 tomato plants for each treatment. All pots were placed in a randomized order and randomly rearranged every 2 days. The disease level was recorded during 0–14 days after the inoculation of strain QL-Rs1115 according to the method of Ciampi-Panno et al. [48], and the disease index was calculated according to the method of Kempe [49]. Based on the trend of the disease index over time, AUDPC was calculated as follows:


$$ AUDPC={\sum}_{i=1}^{n-1}\ \frac{y_i+{y}_{i+1}}{2}\bullet \left({x}_{i+1}-{x}_i\right) $$

*n* is the number of time points.

${y}_i$ and ${y}_{i+1}$ are the disease indices at adjacent time points.

${x}_i$ and ${x}_{i+1}$ are the time intervals between adjacent time points.

### Metabolomic

Biological samples from both ‘Fermented with T-5’ and ‘Fermented without T-5’ treatments were collected, quickly frozen with liquid nitrogen, and stored for subsequent metabolomic analysis. The specific procedure was as follows: 0.5 g of each sample was ground into powder (30 Hz, 1.5 min), 100 mg of the ground sample was weighed and dissolved in 1.2 ml of 70% methanol solution, with the mixture vortexed for 30 s every 30 min, and this process was repeated six times. After centrifugation at 12 000 rpm for 10 min, the supernatant was aspirated, the sample was filtered through a 0.22-μm filter membrane, and the sample was stored in an injection bottle for ultra performance liquid chromatography–tandem mass spectrometry (UPLC-MS/MS) analysis. For the chromatographic analysis, an Agilent SB-C18 column (1.8 μm, 2.1 × 100 mm) was used, with mobile phase A consisting of ultrapure water with 0.1% formic acid and mobile phase B consisting of acetonitrile with 0.1% formic acid. The elution gradient was set as follows: 0.00 min, 5% B; linear increase in B from 5% to 95% within 9 min, then maintained at 95% for 1 min; from 10.00 to 11.10 min, B decreased to 5%, which was maintained at 5% for 14 min, with a flow rate of 0.35 ml/min, column temperature of 40°C, and injection volume of 4 μl. Each analysis was repeated three times. The relative abundance of metabolites in all samples is shown in Table S1.

### Verification of key metabolites

#### Effects of key metabolites on pathogen suppression

A stock solution (20 mM) of each metabolite was prepared by dissolving it in dimethylsulfoxide (DMSO) and filtering the solution through a sterile 0.22-μm filter membrane. To each well of a 96-well microplate, 2 μl of 10% NB medium, 2 μl of stock solution, and 2 μl of *R. solanacearum* seed solution (OD_600_ = 0.5) were sequentially added so that the final concentration of the metabolite was 0.2 mM. Eight replicates were prepared for each metabolite. The stock solution was replaced with an equal volume of pure DMSO (filtered through a sterile 0.22-μm filter membrane) for use as a control. The microplate was then incubated at 170 rpm and 30°C, and the OD_600_ was measured after 48 h to characterize the growth of strain QL-Rs1115.

#### Control effect of key metabolites on tomato wilt disease

A sterile solution of each key metabolite at a final concentration of 0.2 mM per gram of substrate was added to tomato plants by root drenching. An equal volume of sterile water was used as a control. The other operations were the same as in 5.5 and the incidence of tomato wilt under different treatments was recorded.

### Data analysis

Python was used for data analysis. The t-distributed stochastic neighbor embedding was applied to normalized metabolomic data to visualize the differences between treatments using sklearn (v1.2.2) [50], while statistical significance was determined by PERMANOVA using scikit-bio (v0.6.2, https://github.com/scikit-bio/scikit-bio). DI, which characterizes plant residue chemistry, was calculated following the method outlined by Wang et al. [23]. WGCNA, as proposed by Zhang and Horvath, was used to divide metabolites into different modules with PyWGCNA (v2.1.1) [51, 52]. MDT scores of modules were calculated to characterize the importance of each treatment using decoupler-py (v1.8.0) [53], and Mantel correlation between modules and traits was calculated using mantel (https://github.com/jwcarr/mantel). Mann–Whitney *U* test was used for pairwise comparisons between two groups, while Dunn’s test was used for multiple comparisons between multiple groups. *P* < 0.05 was considered statistically significant.

## Acknowledgements

This research was financially supported by the National Key Research and Development Program of China (2023YFD1702200). We would also like to thank TopEdit (www.topeditsci.com) for linguistic assistance during the preparation of this manuscript.

## Supplementary Material

Web_Material_uhaf027

## Data Availability

The data for this article are available in the article or in its supplementary material.
