# Supplementary material for: Fermented chrysanthemum stem as a source of natural phenolic compounds to alleviate tomato bacterial wilt disease
Source: Hortic Res. 2025 Jan 27;12(5):uhaf027. doi: 10.1093/hr/uhaf027 (PMC11986583; doi:10.1093/hr/uhaf027)
Supplement: Web_Material_uhaf027 [file web_material_uhaf027.zip › Supplementary-20250108_clean.docx]

**Supplementary information**

**Fermented chrysanthemum stem as a source of natural phenolic compounds to alleviate tomato bacterial wilt disease**

Peng Ren[^a^](#地址), Peijie Chen[^a^](#地址), Saisai Guo[^a^](#地址), Xinlan Mei[^a^](#地址), Gaofei Jiang[^a^](#地址), Tianjie Yang[^a^](#地址), Xiaofang Wang[^a^](#地址)^,^ [^*^](#通讯), Yangchun Xu[^a^](#地址), Qirong Shen[^a^](#地址), Zhong Wei[^a^](#地址)


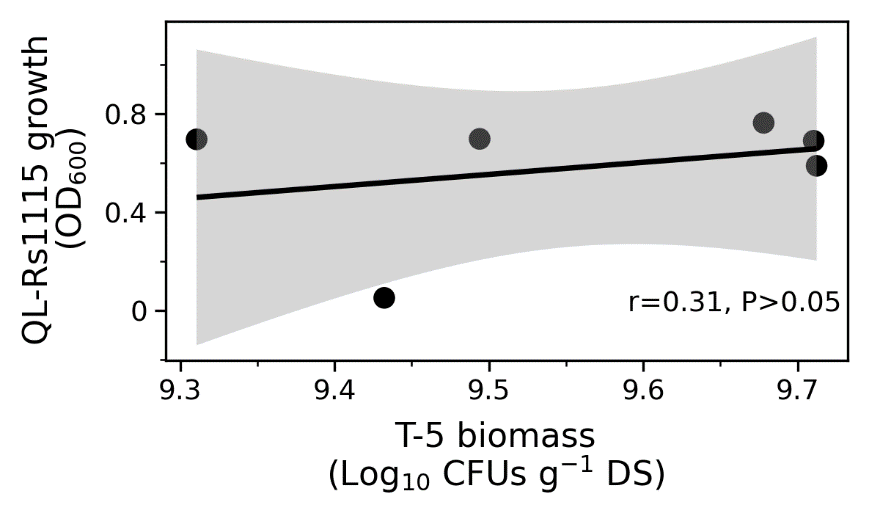


**Fig. S1 Correlation analysis between strain T-5 biomass and QL-Rs1115 growth.** “r” and “*P*” represented Pearson correlation coefficient and significance.


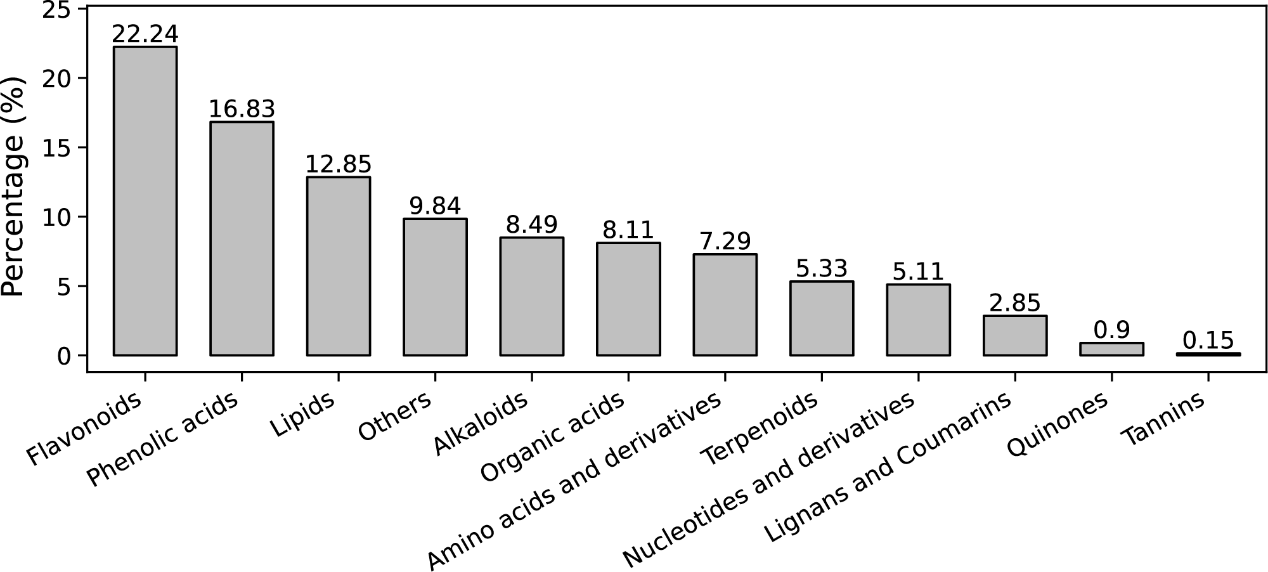


**Fig. S2 Proportion of different metabolite categories.**


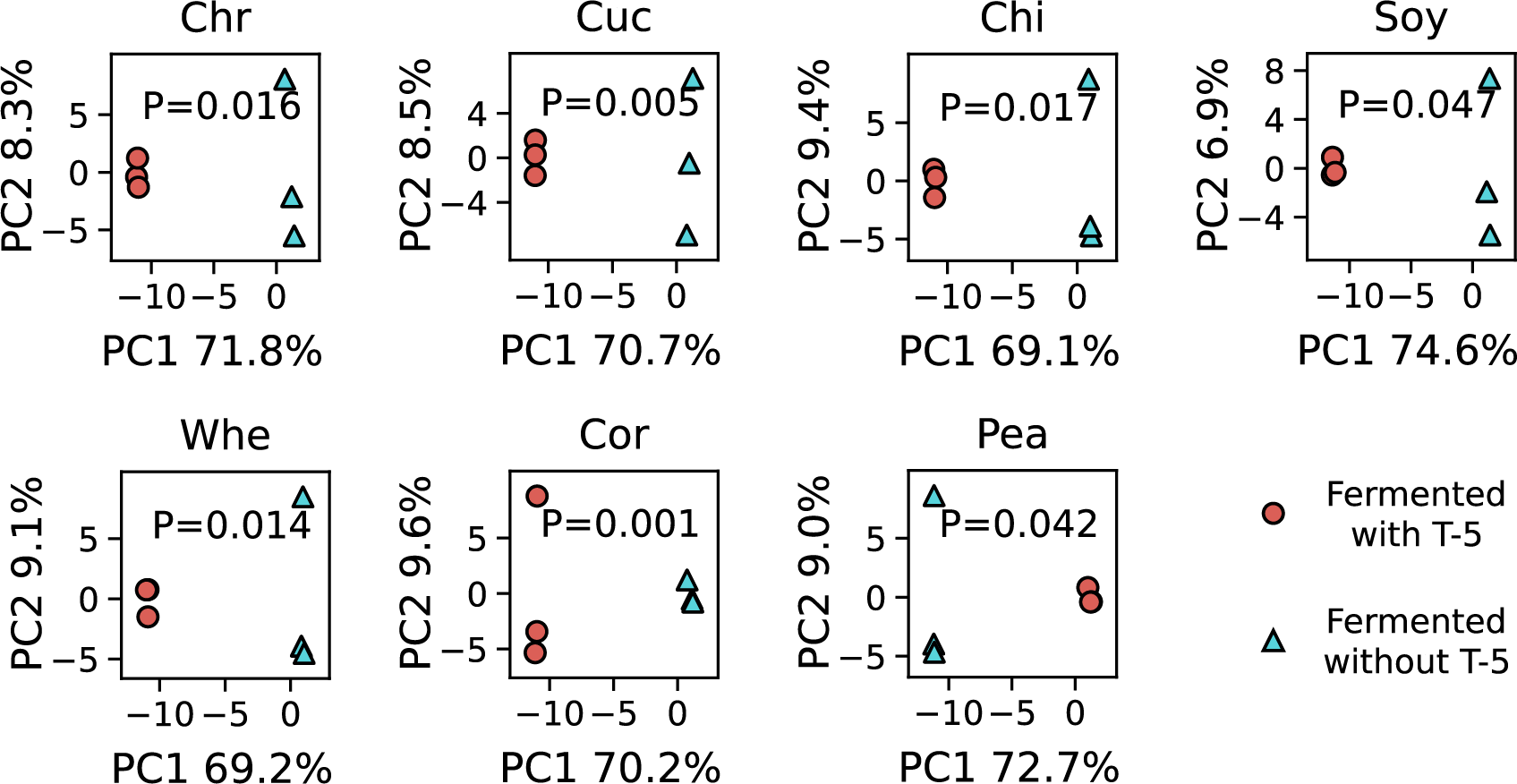


**Fig. S3 Metabolomic difference between each stem fermented with and without strain T-5.** Principal component analysis was used for dimensionality reduction and visualization, and the *P*-value was obtained by PERMANOVA (permutations = 999).


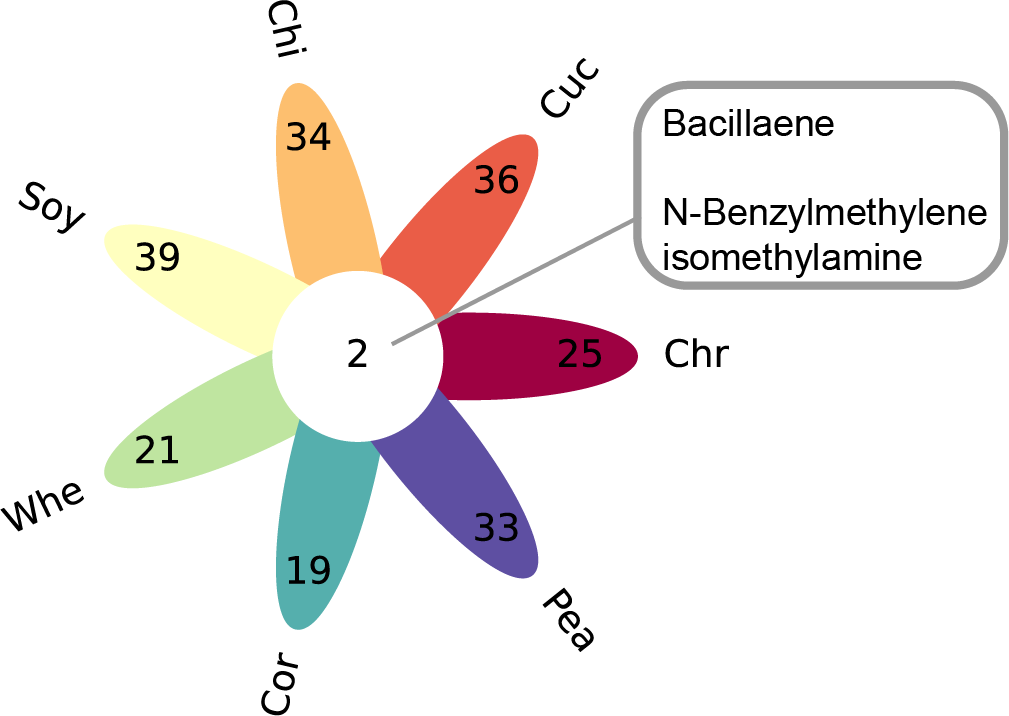


**Fig. S4 Intersection of significantly upregulated SMs.** There were two shared SMs including Bacillaene (Alkaloids) and N-Benzylmethylene isomethylamine (Alkaloids).


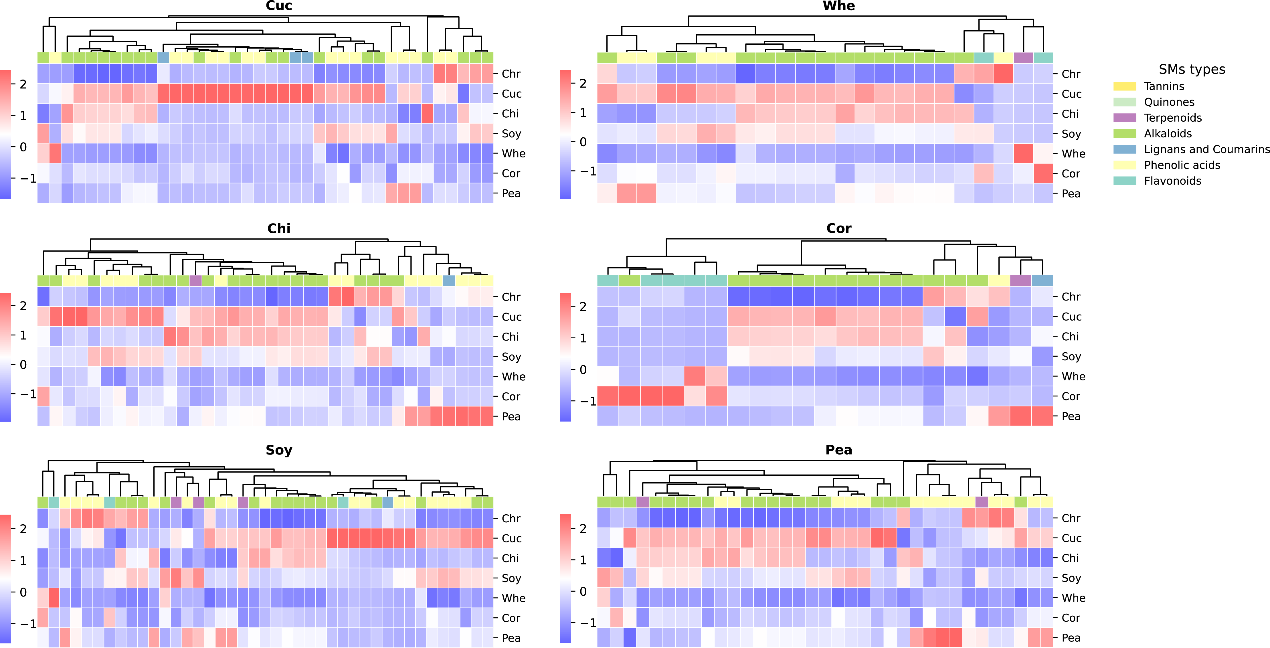


**Fig. S5 Relative abundance comparison of significantly upregulated SMs from stems fermented with strain T-5.**


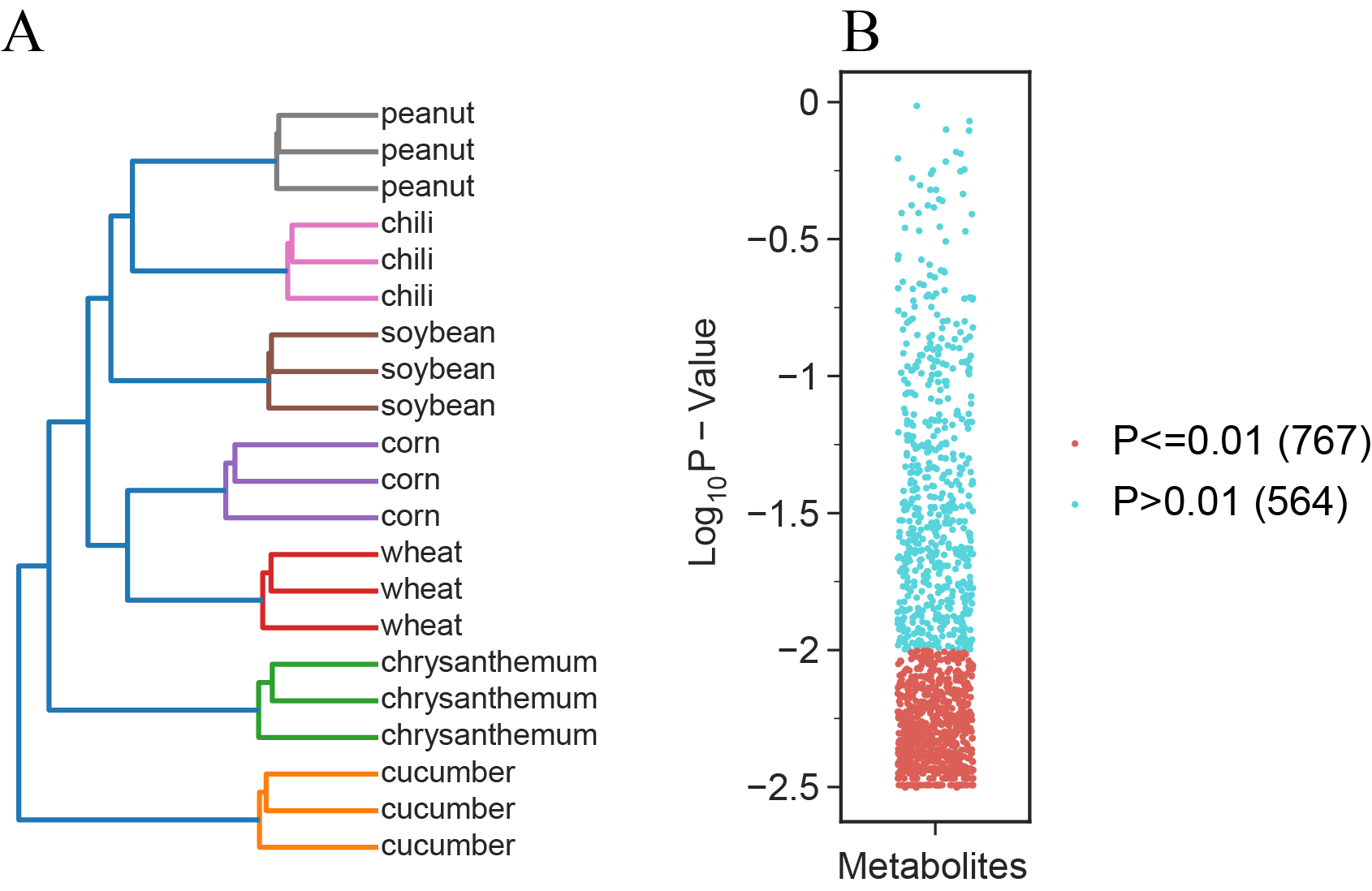


**Fig. S6 Preprocessing of WGCNA.** **A** Hierarchical clustering result of all samples from the “Fermented with T-5” treatment. **B** *P*-values of comparison of each metabolite between groups based on the Kruskal–Wallis test.


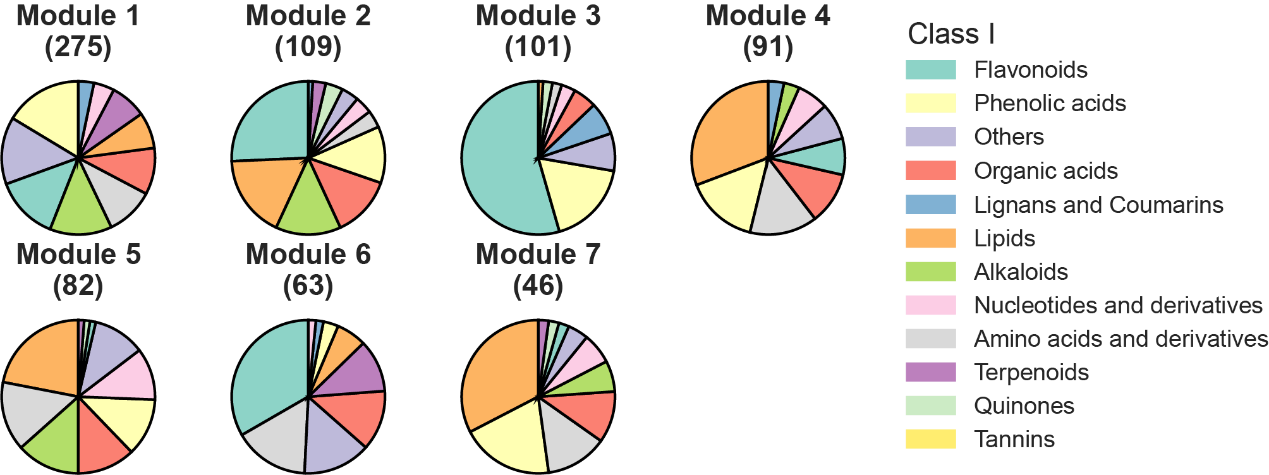


**Fig. S7 Categories of metabolites contained in different modules from WGCNA.** Different colors indicate different categories.


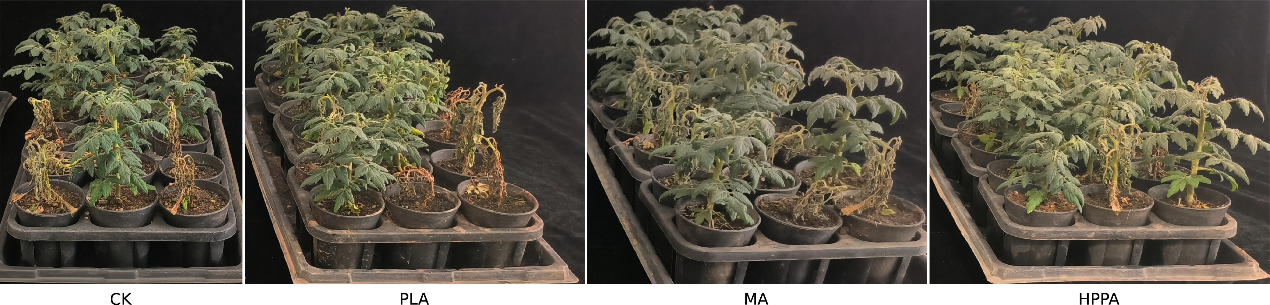


**Fig. S8** **Symptoms of tomato bacterial wilt disease.**

**Table S2 Significantly upregulated differential metabolites of fermented chrysanthemum stem.** The first to fifth columns respectively indicated the names, categories, variable importances in projection, corrected P values based on FDR method and fold changes of the metabolites.

| **Compounds** | **Class I** | **VIP** | **FDR** | **Log2FC** |
| --- | --- | --- | --- | --- |
| Adipic Acid* | Organic acids | 1.24E+00 | 8.78E-04 | 1.58E+00 |
| Bacillaene | Alkaloids | 1.24E+00 | 3.62E-03 | 1.32E+01 |
| β-Hydroxyisovaleric acid | Organic acids | 1.24E+00 | 1.91E-03 | 7.24E+00 |
| Succinyladenosine | Nucleotides and derivatives | 1.24E+00 | 4.22E-03 | 4.39E+00 |
| Methyl L-pyroglutamate | Alkaloids | 1.24E+00 | 2.63E-03 | 2.14E+00 |
| Thymine | Nucleotides and derivatives | 1.24E+00 | 2.63E-03 | 6.54E+00 |
| Hypoxanthine | Nucleotides and derivatives | 1.24E+00 | 6.51E-03 | 5.99E+00 |
| 5'-Deoxy-5'-(methylthio)adenosine | Nucleotides and derivatives | 1.24E+00 | 2.54E-03 | 2.86E+00 |
| L-Phenylalanine | Amino acids and derivatives | 1.24E+00 | 2.49E-03 | 2.03E+00 |
| 3-Isopropylmalic Acid* | Organic acids | 1.24E+00 | 2.39E-03 | 5.96E+00 |
| N6-Isopentenyladenine | Nucleotides and derivatives | 1.24E+00 | 8.96E-03 | 6.16E+00 |
| N-Benzylmethylene isomethylamine | Alkaloids | 1.24E+00 | 6.12E-03 | 1.99E+00 |
| Betaine | Alkaloids | 1.24E+00 | 2.63E-03 | 6.88E+00 |
| D-Pantothenic Acid | Others | 1.24E+00 | 7.84E-04 | 4.31E+00 |
| 2-Hydroxy-3-phenylpropanoic acid | Phenolic acids | 1.24E+00 | 2.01E-03 | 2.94E+00 |
| 2-Isopropylmalic Acid | Organic acids | 1.24E+00 | 4.22E-03 | 6.30E+00 |
| Mandelic acid | Phenolic acids | 1.24E+00 | 7.09E-03 | 2.46E+00 |
| D-Fructose 6-Phosphate | Others | 1.24E+00 | 5.43E-03 | 2.72E+00 |
| LysoPE 15:0(2n isomer) | Lipids | 1.24E+00 | 8.78E-04 | 4.73E+00 |
| 1-Naphthylacetic acid | Organic acids | 1.24E+00 | 5.43E-03 | 7.00E+00 |
| Argininosuccinic acid | Organic acids | 1.24E+00 | 7.68E-03 | 2.64E+00 |
| D-Glucose 6-phosphate* | Others | 1.24E+00 | 7.38E-03 | 2.68E+00 |
| 6-Methylmercaptopurine | Nucleotides and derivatives | 1.24E+00 | 6.86E-03 | 1.96E+00 |
| N6-(2-Hydroxyethyl)adenosine | Nucleotides and derivatives | 1.24E+00 | 4.29E-04 | 1.05E+00 |
| N-Acetylisatin | Alkaloids | 1.24E+00 | 3.83E-03 | 1.86E+00 |
| 2'-O-Methyladenosine | Nucleotides and derivatives | 1.24E+00 | 2.63E-03 | 1.50E+00 |
| 5-O-p-Coumaroylshikimic acid O-glucoside | Phenolic acids | 1.24E+00 | 7.28E-03 | 6.10E+00 |
| Methyl palmitate | Lipids | 1.24E+00 | 8.60E-03 | 3.44E+00 |
| 2-Propylmalic Acid* | Organic acids | 1.24E+00 | 1.06E-06 | 6.01E+00 |
| 3-(4-Hydroxyphenyl)-propionic acid | Phenolic acids | 1.24E+00 | 1.38E-03 | 2.86E+00 |
| Suberic Acid | Organic acids | 1.24E+00 | 2.40E-04 | 2.22E+00 |
| Chrysoeriol-6-C-glucoside-4'-O-glucoside | Flavonoids | 1.24E+00 | 6.09E-04 | 1.28E+00 |
| 8-Azaguanine | Nucleotides and derivatives | 1.24E+00 | 6.71E-04 | 1.05E+00 |
| D-Xylonic acid | Others | 1.24E+00 | 3.20E-03 | 2.14E+00 |
| 2-Dehydro-3-deoxy-L-arabinonate | Others | 1.24E+00 | 8.61E-03 | 3.29E+00 |
| Guanine | Nucleotides and derivatives | 1.24E+00 | 2.35E-03 | 1.11E+00 |
| 2-Methylglutaric acid* | Organic acids | 1.24E+00 | 5.12E-03 | 1.60E+00 |
| 2-Hydroxy-2-methyl-3-oxobutanoic acid | Organic acids | 1.24E+00 | 3.07E-03 | 1.53E+00 |
| 7-Methylguanine | Nucleotides and derivatives | 1.24E+00 | 2.63E-03 | 2.21E+00 |
| L-Alanyl-L-Phenylalanine | Amino acids and derivatives | 1.24E+00 | 5.43E-03 | 4.64E+00 |
| Sorbitol-6-phosphate | Others | 1.24E+00 | 3.62E-03 | 2.53E+00 |
| 3,4-dihydroxy phenylethanol | Phenolic acids | 1.24E+00 | 3.09E-03 | 6.74E+00 |
| Nicotinate D-ribonucleoside | Others | 1.24E+00 | 1.62E-03 | 3.61E+00 |
| 2,5-Dihydroxybenzoic acid; Gentisic Acid* | Phenolic acids | 1.24E+00 | 1.18E-03 | 1.81E+00 |
| cis-4-Hydroxy-D-proline | Amino acids and derivatives | 1.24E+00 | 1.60E-04 | 3.91E+00 |
| 6-O-methylguanine | Nucleotides and derivatives | 1.24E+00 | 3.08E-03 | 2.13E+00 |
| 2-Feruloyl-sn-glycerol | Phenolic acids | 1.24E+00 | 4.31E-04 | 2.24E+00 |
| Hydroxyphenyllactic acid | Phenolic acids | 1.23E+00 | 9.98E-03 | 4.19E+00 |
| 4,5,6-Trihydroxy-2-oxohexanoic acid | Organic acids | 1.23E+00 | 7.02E-03 | 2.32E+00 |
| D-Mandelic acid | Organic acids | 1.23E+00 | 4.56E-03 | 2.57E+00 |
| Evodol | Others | 1.23E+00 | 3.08E-03 | 5.01E+00 |
| Pratensein-7-O-glucoside | Flavonoids | 1.23E+00 | 6.92E-03 | 3.41E+00 |
| D-Galactaric acid* | Others | 1.23E+00 | 5.43E-03 | 2.70E+00 |
| 2,3-Dihydroxybenzoic Acid* | Phenolic acids | 1.23E+00 | 2.63E-03 | 1.69E+00 |
| 3,4-Dihydroxybenzoic acid (Protocatechuic acid)* | Phenolic acids | 1.23E+00 | 1.05E-03 | 1.73E+00 |
| Dehydrodiconiferyl alcohol-gamma'-O-glucoside | Phenolic acids | 1.23E+00 | 9.27E-03 | 1.08E+00 |
| 4-Hydroxyphenylacetic acid | Phenolic acids | 1.23E+00 | 3.57E-03 | 3.32E+00 |
| Methyl 2,4-dihydroxyphenylacetate | Phenolic acids | 1.23E+00 | 1.60E-04 | 4.57E+00 |
| D-Glucoronic acid* | Others | 1.23E+00 | 6.12E-03 | 1.63E+00 |
| Hexanoyl-L-glycine | Amino acids and derivatives | 1.23E+00 | 5.43E-03 | 4.63E+00 |
| Benzoic acid | Phenolic acids | 1.23E+00 | 2.65E-03 | 1.17E+00 |
| Cyclohexylamine | Alkaloids | 1.23E+00 | 2.35E-03 | 1.18E+00 |
| 3-Hydroxyglutaric acid | Organic acids | 1.23E+00 | 7.42E-03 | 2.58E+00 |
| Dimethylmalonic acid* | Organic acids | 1.23E+00 | 2.35E-03 | 1.41E+00 |
| Fraxetin (7,8-Dihydroxy-6-methoxycoumarin) | Lignans and Coumarins | 1.22E+00 | 2.65E-03 | 1.04E+00 |
| N-Acetyl-D-glucosamine-1-phosphate | Others | 1.22E+00 | 2.04E-03 | 2.08E+00 |
| 4-Aminobenzoic acid | Phenolic acids | 1.22E+00 | 7.38E-03 | 1.49E+00 |
| 2-Methylsuccinic acid* | Organic acids | 1.22E+00 | 3.08E-03 | 1.31E+00 |
| 6-Hydroxyhexanoic acid | Organic acids | 1.22E+00 | 7.74E-03 | 2.64E+00 |
| 2-Hydroxyisobutyric acid* | Organic acids | 1.22E+00 | 7.45E-03 | 1.05E+00 |
| Pyrrole-2-carboxylic acid | Organic acids | 1.22E+00 | 4.10E-03 | 1.39E+00 |
| Agmatine | Alkaloids | 1.22E+00 | 4.10E-03 | 1.99E+00 |
| 2-Hydroxyglutaric Acid* | Organic acids | 1.21E+00 | 6.98E-04 | 3.30E+00 |
| L-Cysteine | Amino acids and derivatives | 1.21E+00 | 5.33E-03 | 2.22E+00 |
| 3-Methylmalic acid* | Organic acids | 1.21E+00 | 4.93E-04 | 3.44E+00 |
| 2-Hydroxybutyric Acid* | Organic acids | 1.21E+00 | 7.10E-03 | 1.33E+00 |
| 3-Methyl-2-Oxobutanoic acid | Organic acids | 1.21E+00 | 5.49E-03 | 1.07E+00 |
| Pantetheine | Others | 1.21E+00 | 6.40E-03 | 4.09E+00 |
| 3,4,5-Tricaffeoylquinic acid | Phenolic acids | 1.20E+00 | 5.13E-03 | 1.44E+00 |
| 3-hydroxyphenylacetic acid | Phenolic acids | 1.20E+00 | 1.18E-03 | 3.35E+00 |
| L-Leucyl-L-phenylalanine | Amino acids and derivatives | 1.19E+00 | 1.29E-03 | 4.73E+00 |
| 2-Methyl-3-oxoadipic acid | Organic acids | 1.19E+00 | 5.39E-03 | 2.16E+00 |
| Sebacate | Organic acids | 1.18E+00 | 4.08E-03 | 2.23E+00 |
| L-Citramalic acid | Organic acids | 1.17E+00 | 1.18E-03 | 3.80E+00 |
| 3-Dehydroshikimic acid | Organic acids | 1.15E+00 | 8.79E-03 | 2.79E+00 |
| Pantothenol | Others | 1.15E+00 | 3.72E-03 | 3.10E+00 |
| Lumazine | Nucleotides and derivatives | 1.12E+00 | 1.91E-03 | 4.85E+00 |

**Table S3 Module importance based on multivariate decision tree.** Higher values indicating greater importance.

|  | **Module 1** | **Module 2** | **Module 3** | **Module 4** | **Module 5** | **Module 6** | **Module 7** |
| --- | --- | --- | --- | --- | --- | --- | --- |
| **Chi** | 0.47216 | 0.007888 | 0.012518 | 0.003775 | 0.00752 | 0.00433 | 0.49181 |
| **Chr** | 0.005288 | 0.002508 | 0.960467 | 0.000423 | 0.000376 | 0.000471 | 0.030467 |
| **Cor** | 0.079697 | 0.68713 | 0.004956 | 0.00187 | 0.004586 | 0.002048 | 0.219711 |
| **Cuc** | 0.080555 | 0.000926 | 0.000762 | 0.311185 | 0.227567 | 0.00063 | 0.378376 |
| **Pea** | 0.333076 | 0.006724 | 0.017365 | 0.00705 | 0.069459 | 0.003029 | 0.563298 |
| **Soy** | 0.13003 | 0.024309 | 0.009272 | 0.010577 | 0.035298 | 0.473065 | 0.31745 |
| **Whe** | 0.176732 | 0.408149 | 0.014746 | 0.019084 | 0.009691 | 0.012188 | 0.35941 |

**Table S4 Metabolites in module 3.**

| **Compounds** | **Types** |
| --- | --- |
| 2-hydroxyemodin-1-methylether | Quinones |
| Physcion-8-O-(6-acetyl)-glucoside | Quinones |
| Mandelic acid | Phenolic acids |
| 2-Hydroxy-3-phenylpropanoic acid | Phenolic acids |
| 3-(4-Hydroxyphenyl)-propionic acid | Phenolic acids |
| Caffeic acid | Phenolic acids |
| Syringaldehyde | Phenolic acids |
| Sinapyl alcohol | Phenolic acids |
| Methyl 3-(3-hydroxy-4-methoxyphenyl)propanoate | Phenolic acids |
| Protocatechuic acid-4-O-glucoside* | Phenolic acids |
| 1-O-Gentisoyl-D-glucoside* | Phenolic acids |
| 2,4,6-trihydroxy-acetophenone-6-O-β-D-glucopyranoside | Phenolic acids |
| 3,4,5-Trimethoxyphenyl-1-O-Glucoside | Phenolic acids |
| 2-(3,4-dihydroxyphenyl)ethanediol 1-O-β-D-glucopyranoside | Phenolic acids |
| 3-O-p-Coumaroylquinic acid | Phenolic acids |
| 1-O-p-Coumaroylquinic acid | Phenolic acids |
| Chlorogenic acid (3-O-Caffeoylquinic acid)* | Phenolic acids |
| Cryptochlorogenic acid (4-O-Caffeoylquinic acid) | Phenolic acids |
| 4-O-Glucosyl-sinapate | Phenolic acids |
| Isochlorogenic acid B | Phenolic acids |
| 5,7-Dihydroxychromone | Others |
| D-Fructose 6-Phosphate | Others |
| D-Glucose-1-phosphate* | Others |
| D-Glucose 6-phosphate* | Others |
| D-Glucose 1,6-bisphosphate | Others |
| Majoroside | Others |
| Raffinose | Others |
| Maltotriose | Others |
| Muconic acid | Organic acids |
| Mevalonic acid | Organic acids |
| 2-Oxoadipic acid | Organic acids |
| Phosphoenolpyruvate | Organic acids |
| DL-Glyceraldehyde-3-phosphate | Organic acids |
| Isocytosine | Nucleotides and derivatives |
| 2'-Deoxyinosine-5'-monophosphate | Nucleotides and derivatives |
| Succinyladenosine | Nucleotides and derivatives |
| Methyl linolenate | Lipids |
| Esculetin | Lignans and Coumarins |
| 6,7-Dihydroxy-4-methylcoumarin | Lignans and Coumarins |
| Esculin (6,7-DihydroxyCoumarin-6-glucoside) | Lignans and Coumarins |
| Daphnin | Lignans and Coumarins |
| Syringic acid 4-O-rhamnoside | Lignans and Coumarins |
| Pinoresinol-4-O-glucoside | Lignans and Coumarins |
| 1-Hydroxypinoresinol-1-O-Glucoside | Lignans and Coumarins |
| Luteolin (5,7,3',4'-Tetrahydroxyflavone) | Flavonoids |
| Rhamnocitrin (7-Methylkaempferol)* | Flavonoids |
| Hispidulin (5,7,4'-Trihydroxy-6-methoxyflavone)* | Flavonoids |
| Pratensein | Flavonoids |
| Diosmetin (5,7,3'-Trihydroxy-4'-methoxyflavone) | Flavonoids |
| 4',5,7-Trihydroxy-3',6-dimethoxyflavone (Jaceosidin) | Flavonoids |
| Eupatilin (5,7-Dihydroxy-3',4',6-Trimethoxyflavone) | Flavonoids |
| 5-Hydroxy-6,7,3',4'-tetramethoxyflavone | Flavonoids |
| Eupatorin-5-methylether (3'-hydroxy-5,6,7,4'-tetramethoxyflavone) | Flavonoids |
| Apigenin-7-O-glucoside(Cosmosiin) | Flavonoids |
| Apigenin-7-O-glucuronide | Flavonoids |
| Trifolirhizin (Maackiain-3-O-glucoside) | Flavonoids |
| Acacetin-7-O-glucoside (Tilianin)* | Flavonoids |
| Kaempferol-3-O-galactoside (Trifolin) | Flavonoids |
| Luteolin-7-O-glucoside (Cynaroside) | Flavonoids |
| Eriodictyol-3'-O-glucoside | Flavonoids |
| Acacetin-7-O-glucuronide | Flavonoids |
| Luteolin-7-O-glucuronide | Flavonoids |
| Kaempferol-3-O-glucuronide | Flavonoids |
| 6-C-MethylKaempferol-3-glucoside | Flavonoids |
| Diosmetin-7-O-galactoside* | Flavonoids |
| Diosmetin-7-O-glucoside* | Flavonoids |
| Hesperetin-5-O-glucoside | Flavonoids |
| Isorhamnetin-3-O-gallate | Flavonoids |
| Apigenin-7-O-(6''-acetyl)glucoside | Flavonoids |
| Diosmetin-7-O-glucuronide | Flavonoids |
| 5,2'-Dihydroxy-7,8-dimethoxyflavone glycosides | Flavonoids |
| Quercetin-4'-O-glucuronide | Flavonoids |
| Isorhamnetin-3-O-Glucoside* | Flavonoids |
| Isorhamnetin-7-O-glucoside (Brassicin)* | Flavonoids |
| Rhamnetin-3-O-Glucoside | Flavonoids |
| Nepetin-7-O-glucoside | Flavonoids |
| Kaempferol-3-O-(2''-acetyl)glucoside | Flavonoids |
| Tricin-7-O-Glucuronide | Flavonoids |
| Apigenin-6,8-di-C-arabinoside | Flavonoids |
| Chrysoeriol-7-O-(6''-malonyl)glucoside | Flavonoids |
| Tricin-7-O-(6''-O-malonyl)glucoside | Flavonoids |
| Apigenin-7-O-(6''-p-Coumaryl)glucoside | Flavonoids |
| Naringenin-7-O-Neohesperidoside(Naringin)* | Flavonoids |
| Naringenin-7-O-Rutinoside(Narirutin)* | Flavonoids |
| Chrysoeriol-6-C-rhamnoside-7-O-rhamnoside | Flavonoids |
| Acacetin-7-O-rutinoside (Linarin) | Flavonoids |
| Luteolin-7-O-rutinoside* | Flavonoids |
| Kaempferol-3-O-neohesperidoside | Flavonoids |
| Poncirin (Isosakuranetin-7-O-neohesperidoside) | Flavonoids |
| Eriodictyol-7-O-Rutinoside (Eriocitrin) | Flavonoids |
| Diosmetin-7-O-Neohesperidoside (Neodiosmin)* | Flavonoids |
| Diosmetin-7-O-rutinoside (Diosmin)* | Flavonoids |
| Chrysoeriol-7-O-rutinoside | Flavonoids |
| Hispidulin-7-O-(6''-O-p-Coumaroyl)Glucoside | Flavonoids |
| Hispidulin-8-C-(2''-O-glucosyl)glucoside | Flavonoids |
| Chrysoeriol-7-O-gentiobioside | Flavonoids |
| Chrysoeriol-6-C-glucoside-4'-O-glucoside | Flavonoids |
| Hesperidin methylchalcone | Flavonoids |
| Isorhamnetin-3-O-sophoroside | Flavonoids |
| N-Propionylglycine | Amino acids and derivatives |
| S-Ribosyl-L-homocysteine | Amino acids and derivatives |

**Table S5 Prediction of secondary metabolites from *Bacillus amyloliquefaciens* T-5.** Complete genome of *Bacillus amyloliquefaciens* T-5 (NCBI Accession: PRJNA660531) was using to predict secondary metabolites on antiSMASH platform (v7.0.0). 13 genome regions were obtained, with metabolites (significant hits) listed. For duplicate metabolites, only the metabolite with the highest "Cumulative BLAST score" was retained.

| **Region** | **Secondary metabolites** | **Cumulative BLAST score** |
| --- | --- | --- |
| Region7 | bacillaene | 46660 |
| Region8 | fengycin | 46118 |
| Region11 | difficidin | 44526 |
| Region6 | macrolactin H | 34762 |
| Region6 | macrolactin H/macrolactin B/macrolactin 1c/macrolactin E | 34029 |
| Region8 | bacillomycin D | 31887 |
| Region8 | iturin | 30574 |
| Region8 | plipastatin | 29681 |
| Region2 | surfactin | 29000 |
| Region8 | mycosubtilin | 26874 |
| Region8 | paenilarvin A/paenilarvin B/paenilarvin C | 21706 |
| Region2 | lichenysin | 13444 |
| Region8 | paenibacterin | 11898 |
| Region8 | brevicidine | 9526 |
| Region8 | tridecaptin M | 9358 |
| Region12 | bacillibactin | 7525 |
| Region8 | octapeptin C4 | 5706 |
| Region12 | bacillibactin/bacillibactin E/bacillibactin F | 5407 |
| Region7 | aurantinin B/aurantinin C/aurantinin D | 5329 |
| Region12 | paenibactin | 5127 |
| Region13 | bacilysin | 4636 |
| Region7 | macrobrevin | 3992 |
| Region11 | basiliskamide A/basiliskamide B | 3958 |
| Region11 | elansolid A1 | 3581 |
| Region12 | griseobactin | 3547 |
| Region12 | benarthin/dibenarthin | 3285 |
| Region11 | swinholide A | 3184 |
| Region11 | lagriamide | 3184 |
| Region11 | kalimantacin A | 3160 |
| Region1 | locillomycin/locillomycin B/locillomycin C | 3140 |
| Region7 | gladiostatin A | 2897 |
| Region6 | chivosazol | 2857 |
| Region11 | oocydin A | 2620 |
| Region7 | scytophycin | 2586 |
| Region7 | misakinolide A | 2551 |
| Region12 | amylocyclicin | 2384 |
| Region7 | calyculin A/phosphocalyculin A/calyculinamide A/dephosphonocalyculin A | 2373 |
| Region7 | myxovirescin A1 | 2349 |
| Region7 | dumulmycin/shuangdaolide A/shuangdaolide C/shuangdaolide B/shuangdaolide D | 2262 |
| Region11 | lagriene | 2131 |
| Region11 | sorangicin A | 2088 |
| Region7 | pseudomonic acid A | 2080 |
| Region12 | lipopeptide 8D1-1/lipopeptide 8D1-2 | 2051 |
| Region7 | thiomarinol | 2041 |
| Region7 | mycalamide A | 2010 |
| Region12 | CDA1b/CDA2a/CDA2b/CDA3a/CDA3b/CDA4a/CDA4b | 1988 |
| Region12 | hormaomycin/hormaomycin A1/hormaomycin A2/hormaomycin A3/hormaomycin A4/hormaomycin A5/hormaomycin A6 | 1985 |
| Region12 | marformycin A/marformycin B/marformycin C/marformycin D/marformycin E/marformycin F | 1982 |
| Region11 | luminaolide | 1955 |
| Region7 | phormidolide | 1934 |
| Region7 | pyxipyrrolone A/pyxipyrrolone B | 1911 |
| Region12 | acyldepsipeptide 1 | 1907 |
| Region12 | myxochelin B/myxochelin N/myxochelin O/myxochelin P/myxochelin Q/myxochelin A | 1888 |
| Region12 | glycinocin A | 1850 |
| Region12 | actinomycin D | 1841 |
| Region12 | enduracidin | 1781 |
| Region7 | leptolyngbyalide | 1715 |
| Region7 | sesbanimide C/sesbanimide F/sesbanimide E | 1714 |
| Region7 | cusperin | 1706 |
| Region12 | pepticinnamin E | 1684 |
| Region7 | lasonolide A | 1612 |
| Region11 | thailandamide/thailandamide lactone | 1569 |
| Region12 | aminochelin/azotochelin/protochelin | 1556 |
| Region12 | amphi-enterobactin 1/amphi-enterobactin 2/amphi-enterobactin 3/amphi-enterobactin 4 | 1554 |
| Region7 | isobongkrekic acid/bongkrekic acid | 1548 |
| Region7 | pulvomycin B/pulvomycin C/pulvomycin D | 1547 |
| Region7 | labrenzin | 1522 |
| Region12 | bonnevillamide D/bonnevillamide E | 1508 |
| Region11 | FR901464 | 1492 |
| Region7 | corallopyronin | 1429 |
| Region7 | thailanstatin A | 1416 |
| Region7 | spliceostatin/FR901464 | 1416 |
| Region7 | gynuellalide | 1395 |
| Region7 | psymberin/irciniastatin B | 1349 |
| Region6 | gladiofungin A/gladiofungin B | 1329 |
| Region12 | fuscachelin A/fuscachelin B/fuscachelin C | 1272 |
| Region8 | corynecin III/corynecin I/corynecin II | 1257 |
| Region7 | nosperin | 1244 |
| Region12 | myxochelin B/myxochelin A/pseudochelin A | 1217 |
| Region7 | iso-migrastatin/migrastatin/dorrigocin A/dorrigocin B/13-epi-dorrigocin A | 1207 |
| Region7 | lactimidomycin/8,9-dihydrolactimidomycin/8-hydroxy-8,9-dihydrolactidomycin/17-hydroxy-8-desmethoxy-isomigrastatin | 1158 |
| Region7 | spliceostatin L/spliceostatin M | 1152 |
| Region7 | ripostatin A/ripostatin B/ripostatin C | 1140 |
| Region12 | mirubactin | 1101 |
| Region12 | 2,3-dihydroxybenzoylserine | 1099 |
| Region11 | largimiycin 38/largimycin 39 | 1091 |
| Region3 | butirosin A/butirosin B | 1038 |
| Region12 | frederiksenibactin | 1034 |
| Region12 | caboxamycin | 1032 |
| Region12 | amonabactin P 750 | 1008 |
| Region12 | nataxazole | 997 |
| Region7 | cylindrocyclophane D/cylindrocyclophane E/cylindrocyclophane F | 966 |
| Region7 | cylindrocyclophane | 966 |
| Region7 | carbamidocyclophane A/carbamidocyclophane B/carbamidocyclophane C/carbamidocyclophane D/carbamidocyclophane E/carbamidocyclophane F/carbamidocyclophane H/carbamidocyclophane M/carbamidocyclophane N/carbamidocyclophane O/carbamidocyclophane P/carbamidocyclophane Q/carbamidocyclophane R/carbamidocyclophane S/carbamidocyclophane T/carbamidocyclophane U | 965 |
| Region12 | obafluorin | 939 |
| Region7 | apratoxin A | 934 |
| Region7 | vatiamide A/vatiamide B/vatiamide C/vatiamide D/vatiamide E/vatiamide F | 926 |
| Region7 | jamaicamide A/jamaicamide B/jamaicamide C | 922 |
| Region12 | parabactin | 910 |
| Region7 | malyngamide I | 897 |
| Region11 | palmerolide D/palmerolide H | 896 |
| Region11 | palmerolide A/palmerolide B/palmerolide C/palmerolide F/palmerolide G | 896 |
| Region13 | S-layer glycan | 895 |
| Region7 | pederin | 893 |
| Region12 | heterobactin B/heterobactin S2 | 886 |
| Region12 | heterobactin A/heterobactin S2 | 883 |
| Region7 | bryostatin | 881 |
| Region12 | vibriobactin | 843 |
| Region1 | bacinapeptin | 810 |
| Region12 | vanchrobactin | 804 |
| Region12 | chromobactin | 791 |
| Region12 | enterobactin | 787 |
| Region12 | vulnibactin | 780 |
| Region12 | agrobactin | 772 |
| Region12 | photobactin | 766 |
| Region12 | A33853 | 751 |
| Region1 | andalusicin A/andalusicin B | 692 |
| Region8 | lipopolysaccharide | 535 |
| Region12 | streptonigrin | 509 |
| Region3 | gramicidin S | 500 |
| Region12 | diazaquinomycin H/diazaquinomycin J | 491 |
| Region12 | diazaquinomycin A/diazaquinomycin E/diazaquinomycin F/diazaquinomycin G | 486 |
| Region12 | paulomycin | 482 |
| Region12 | murayaquinone | 471 |
| Region8 | paramagnetoquinone 1/paramagnetoquinone 2 | 455 |
| Region12 | limazepine C/limazepine D/limazepine E/limazepine F/limazepine A | 407 |
| Region12 | fimsbactin A | 392 |
| Region13 | ECO-02301 | 351 |
| Region13 | rubradirin | 339 |
